# White Light, Magnifying Endoscopy, Endocytoscopy, and Artificial Intelligence in Diagnosis of Early Colorectal Cancer: A Comparative Study

**DOI:** 10.1002/deo2.70240

**Published:** 2025-11-17

**Authors:** Eri Tamura, Shin‐ei Kudo, Shunto Iwasaki, Shigenori Semba, Tomoya Shibuya, Shun Kato, Takanori Kuroki, Yuta Sato, Tatsuya Sakurai, Yushi Ogawa, Yuta Kouyama, Yasuharu Maeda, Katsuro Ichimasa, Noriyuki Ogata, Takemasa Hayashi, Kunihiko Wakamura, Hideyuki Miyachi, Toshiyuki Baba, Fumio Ishida, Tetsuo Nemoto, Masashi Misawa

**Affiliations:** ^1^ Digestive Disease Center Showa Medical University Northern Yokohama Hospital Kanagawa Japan; ^2^ Department of Gastroenterology and Hepatology Kochi Medical School Kochi University Kochi Japan; ^3^ Satoh Clinic Saga Japan; ^4^ Department of Diagnostic Pathology Showa Medical University Northern Yokohama Hospital Kanagawa Japan

**Keywords:** artificial intelligence, colorectal neoplasms, computer‐aided diagnosis, endoscopy

## Abstract

**Objectives:**

Early detection of colorectal cancer is critical for improving prognosis. However, assessing invasion depth—distinguishing between superficial cancer (T1a) and deep submucosal invasive cancer (T1b)—remains challenging. Recently, artificial intelligence (AI)‐assisted computer‐aided diagnosis (CADx) systems have been introduced to complement conventional endoscopy. This study aims to compare the diagnostic accuracy of endoscopists in predicting deep submucosal invasion in early colorectal cancer under four modalities: white‐light imaging (WLI), magnifying endoscopy (including narrow‐band imaging magnification and pit pattern), endocytoscopy (EC), and CADx support.

**Methods:**

We conducted a single‐center retrospective study using stored endoscopic images between April 2021 and December 2022. Each lesion was evaluated using white light imaging, magnifying endoscopy, EC, and CADx analysis with the EndoBRAIN‐Plus system. Trainee and expert endoscopists assessed the images sequentially, recording their estimations of invasion depth (T1a vs. T1b) and confidence levels. Sensitivity, specificity, and accuracy were calculated against the pathological reference. We compared performances stratified by confidence level and endoscopist experience.

**Results:**

During the study period, 66 lesions were eligible. Of them, 27% (18 lesions) were T1b cancers. Diagnostic accuracy improved progressively from white light imaging (82.7% [95% confidence interval {95%CI}: 81.2–86.9]) to EC (85.6% [95%CI: 82.7–88.2]). The highest specificity and accuracy were achieved when AI‐assisted diagnosis was incorporated (accuracy: 88.9% [95%CI: 86.3–91.2], specificity: 93.1% [95%CI: 90.6–95.2]). The proportion of high‐confidence readings rose from 40.2% to 75.5%. This was most pronounced in the trainee group.

**Conclusions:**

Integrating advanced endoscopic imaging with CADx significantly improved accuracy in assessing invasion depth. This approach may guide treatment decisions in early‐stage colorectal cancer.

## 1 Introduction

In recent years, colorectal cancer has been the third most common cause of cancer mortality worldwide [1]. Because these cancers have a relatively good prognosis if detected at an early stage [2], early detection and treatment through regular colonoscopies is ideal. Endoscopic treatment alone can cure early‐stage colorectal cancer when no pathological high‐risk factors are present. Therefore, accurate assessment of the depth of early colorectal cancer (cancer invasion deeper than the deep submucosal layer [>1000 µm or not]) is important in planning optimal treatment.

The gold standard for assessment of depth of invasion is the diagnosis of pit pattern by performing magnifying endoscopy under crystal violet staining. In colorectal lesions, this procedure can diagnose V_I_ pit patterns. These patterns distinguish between cancer invasion of more versus less than 1000 µm, the former requiring surgery, whereas endoscopic mucosal resection/endoscopic submucosal resection (EMR/ESD) is recommended for the latter [3]. Recently, endocytoscopy (EC) [4, 5] and artificial intelligence (AI) software (computer‐aided diagnosis [CADx]) have been introduced into clinical practice [6, 7]. It has been reported that EC confers additional diagnostic value to pit pattern diagnosis in assessing invasion of the deep submucosal layers [8]. However, to the best of our knowledge, there are no published studies comparing white light and magnifying endoscopy diagnosis.

This study aims to compare the diagnostic accuracy of endoscopists in predicting deep submucosal invasion in early colorectal cancer under four modalities: white‐light imaging (WLI), magnifying endoscopy (pit pattern and narrow‐band imaging [NBI] magnification), EC, and CADx support.

## 2 Methods

### 2.1 Study Design

This was a single‐center retrospective study. We created an image‐reading test using stored still images obtained from April 2021 to December 2022 at Showa Medical University Northern Yokohama Hospital, Yokohama, Japan.

### 2.2 Lesions

The lesions studied were in a consecutive series of patients who had undergone magnifying endoscopy (NBI and pit pattern), EC examination, and CADx diagnosis before treatment. The inclusion criteria were: (i) pathologically confirmed lesion; and (ii) lesions for which WLI, magnifying endoscopy, EC, and CADx diagnoses had been obtained. The exclusion criteria were: (i) inflammatory bowel disease; (ii) lesions other than adenoma and early colorectal cancer (including sessile serrated lesions [SSLs] and hyperplastic polyps); and (iii) refusal to participate in this study. In our institution, we perform EC examinations when cancer is suspected, before ESD treatment, or when a definitive diagnosis is difficult with magnifying endoscopy.

### 2.3 Pathological Diagnosis

After endoscopic or surgical resection, all specimens were fixed in formalin. The formalin‐fixed specimens were then sectioned and subjected to diagnostic evaluation by gastroenterological pathologists. Pathological diagnosis of all lesions was in accordance with the 2019 World Health Organization classification [9]. Submucosal invasive cancers were divided into two categories, namely, deep (≥1000 µm) submucosal invasive cancer (T1b) and shallow (<1000 µm) submucosal invasive cancer (T1a).

### 2.4 Endoscopic Equipment and Image Acquisition Protocol

**FIGURE 1:**
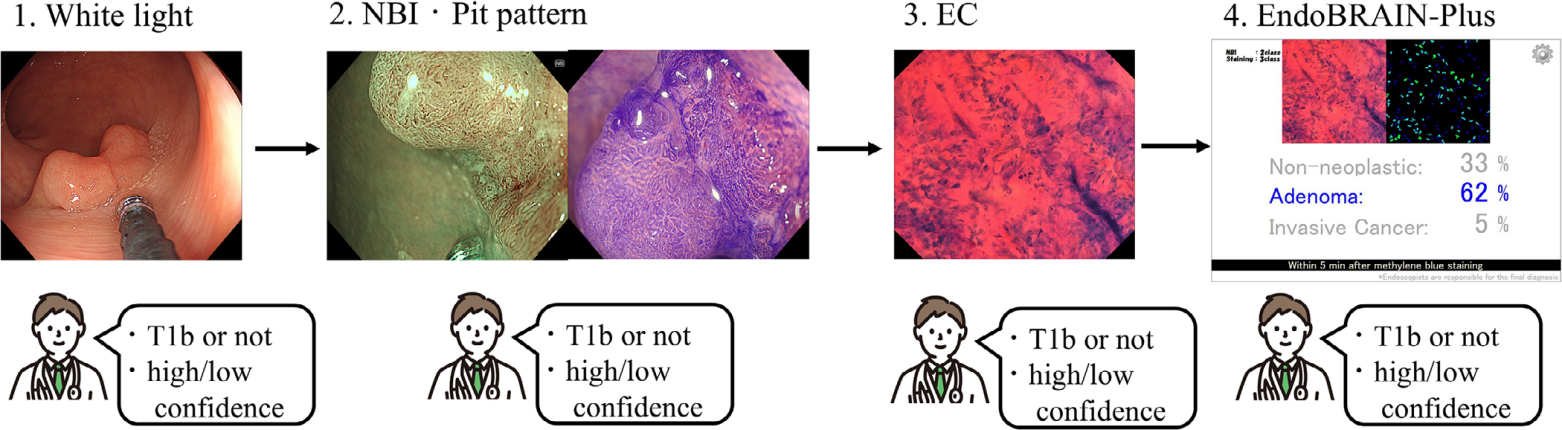
Endoscopic equipment and image acquisition protocol (1) imaging; (2) magnifying narrow‐band imaging and stained with crystal violet; (3) stained with 1% methylene blue; and (4) artificial intelligence (AI) diagnosis.

We used high‐definition video endoscope systems (EVIS LUCERA ELITE and EVIS X1 systems; Olympus, Tokyo, Japan) and high‐vision endoscopes (CF‐XZ1200, CF‐EZ1500, PCF‐H290Z, and CF‐H290ECI; Olympus) in this study. Upon detection, all lesions were washed with water and examined using WLI and magnifying NBI. Following magnifying NBI examination, the lesions were stained with crystal violet for pit pattern diagnosis. If the lesions were suspected to be cancerous, the endoscopists conducted EC examinations at their discretion. If any scope other than the CF‐H290ECI had been in use, endoscopists removed it and inserted a CF‐H290ECI at the lesion site. Subsequently, the lesions were stained with 1% methylene blue for contrast examination of cell nuclei. After dye staining, we obtained in vivo cellular images using the full magnification power of the CF‐H290ECI (Figure 1).

### 2.5 Overview of the AI Software in this Study

In this study, we employed EndoBRAIN‐Plus (Cybernet System; Tokyo, Japan), which is regulatory‐approved CADx software. CADx software is designed to analyze EC images. CADx can output three class predictions: non‐neoplastic, adenoma, and invasive cancer. We have reported details of the algorithm in a previous study [10]. All AI‐generated diagnoses are automatically saved to the electronic medical record server of the endoscopy system.

### 2.6 Details of the Image‐Reading Test

We extracted one endoscopic image for each of the following: WLI, magnifying endoscopy, and EC. We also extracted three AI predictions. Each image was individually examined by 10 endoscopists (five trainees and five experts: We defined “trainee” as a senior resident, and those above that level as “expert”.) in the following sequence: WLI, magnifying endoscopy, EC, and CADx. The endoscopists diagnosed whether the depth of the lesion was <1000 µm (T1a) or deeper than 1000 µm (T1b) and were asked to record their confidence level (high or low). We defined a high‐confidence diagnosis as one in which the physician considered the diagnosis to be correct with approximately 90% probability. This definition has also been adopted in previous studies [11, 12]. The endoscopists’ diagnoses and confidence levels for each modality were recorded and subsequently compared with the final pathological diagnosis. All endoscopists interpreted the images independently and were fully blinded to the reference‐standard pathological diagnoses.

### 2.7 Outcome Measurements

The primary outcome of this study was the diagnostic performance of the endoscopists. The sensitivity, specificity, and accuracy of endoscopists were calculated based on the findings with each modality. The secondary outcomes were to compare the sensitivity, specificity, and accuracy between high and low confidence levels.

### 2.8 Statistical Analysis

Sensitivity, specificity, and accuracy were calculated by comparing the endoscopists’ and pathological diagnoses. We also compared the diagnostic performances between experts and trainees. We compare paired proportions (e.g., accuracies) between modalities using McNemar's test. All *p*‐values are two‐sided, and *p* < 0.05 was considered to denote statistical significance. When we stratified diagnostic accuracy by confidence levels, resulting in an unpaired test, we employed Fisher's exact test. We applied the Bonferroni correction to pairwise comparisons among the four modalities: WLI, magnifying endoscopy, EC, and AI. The Fleiss Kappa value was calculated to evaluate the agreement rate among three or more examiners. The agreement rate was as follows: <0: poor, 0–0.20: slight, 0.21–0.40: fair, 0.41–0.60: moderate, 0.61–0.80: substantial, and 0.81–1.00: almost perfect. All statistical analyses were performed using EZR (Saitama Medical Center, Jichi Medical University, Saitama, Japan), a graphical user interface for R (version 4.2.2; The R Foundation for Statistical Computing, Vienna, Austria) [13]. This was a pilot study, meaning that no reference for accuracy was available. Thus, we could not calculate the required sample size.

### 2.9 Ethics

This study was approved by the Ethics Committee of Showa University (No. 22‐191‐B). The opportunity to refuse to participate in the study was guaranteed using an opt‐out method, meaning it was not necessary to obtain direct written consent from the patients. With an opt‐out approach, failure to explicitly refuse the secondary use of data is considered consent. While obtaining their consent for colonoscopy, we informed all patients about the possibility of using secondary data. If a patient declined to participate in the study or did not consent to secondary use of data, their data were excluded from our analysis. Consent from the endoscopists was obtained in writing.

## 3 Results

### 3.1 Characteristics of Test Images

**FIGURE 2:**
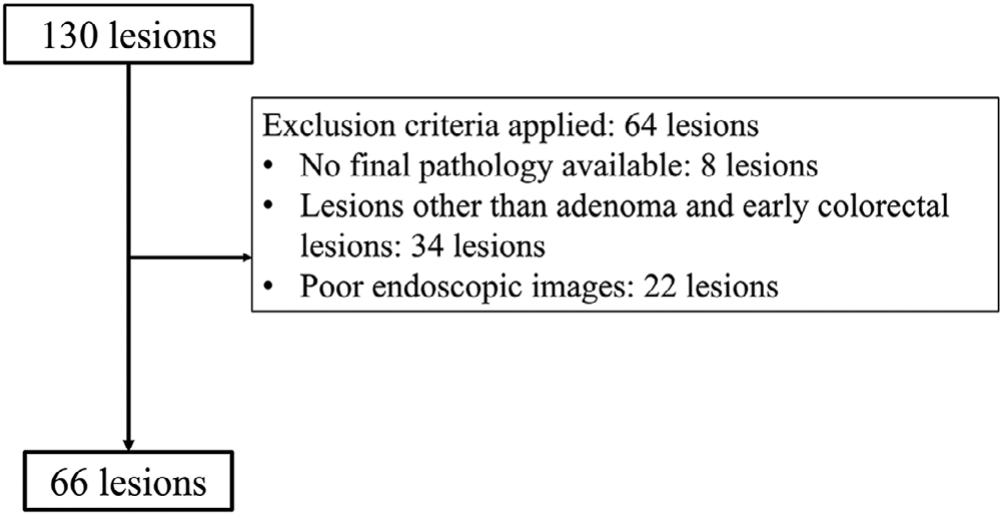
During the study period, 130 lesions were diagnosed with the assistance of EndoBRAIN‐Plus and were removed in the study hospital. Sixty‐six of these lesions were excluded in accordance with the study criteria.

During the study period, 130 lesions were diagnosed with the assistance of CADx. Sixty‐six of these lesions met the inclusion and exclusion criteria and were included in the analysis (Figure 2). Table 1 shows the characteristics of these lesions. They comprised 20 adenomas, 26 intramucosal cancers, two T1a cancers, and 18 T1b cancers.

**TABLE 1:** Characteristics of the included lesions.

Lesion, *n*	66
Patients, *n*	66
Sex (Male, Female), *n*	34:32
Mean age (years), (IQR)	68 (58–79)
Mean lesion size (mm), (IQR)	22.8 (15–30)
Lesion location, *n* (%)	
Right side	32 (48)
Left side	17 (26)
Rectum	17 (26)
Macroscopic appearance, *n* (%)	
Protruding	21 (32)
Flat	37 (56)
Depressed	8 (12)
Pathology, *n* (%)	
Adenoma	20 (30)
Tis	26 (40)
T1a	2 (3)
T1b	18 (27)

Abbreviation: IQR, interquartile range.

### 3.2 Diagnostic Performance

Table 2 shows the diagnostic performances of all participating endoscopists for high confidence and regardless of their level of confidence. The endoscopists diagnosed 265, 418, 473, and 498 lesions with high confidence when using WLI, NBI/pit, EC, and AI, respectively. Sensitivity did not differ significantly between the diagnostic modalities. The highest level of specificity was achieved by the output of AI (with CADx), followed by EC, magnifying endoscopy, and WLI.

**TABLE 2:** Diagnostic performances of all endoscopists: All cases versus high confidence cases.

*n* = 660	WLI		NBI/pit		EC		AI	
Confidence level	All	High	All	High	All	High	All	High
Sensitivity (%) [95% CI]	83.9 [77.7–88.9]	93.9 [85.2–98.3]	78.9 [72.2–84.6]	90.8 [82.7–95.9]	75.0 [68.0–81.1]	81.6 [72.7–88.5]	77.8 [71.0–83.6]	89.8 [82.5–94.8]
Specificity (%) [95% CI]	84.4 [80.8–87.5]	93.0 [88.5–96.1]	86.9 [83.5–89.8]	93.4 [90.1–95.8]	89.6 [86.5–92.2]	95.9 [93.4–97.7]	93.1 [90.6–95.2]	96.9 [94.7–98.4]
Accuracy (%) [95% CI]	82.7 [81.2–86.9]	93.2 [89.5–95.9]	84.7 [81.7–87.4]	92.8 [89.9–95.1]	85.6 [82.7–88.2]	92.8 [90.1–95.0]	88.9 [86.3–91.2]	95.4 [93.2–97.1]

Abbreviations: AI, artificial intelligence; CI, confidence interval; EC, endocytoscopy; NBI, narrow‐band imaging; WLI, white light imaging.

**FIGURE 3:**
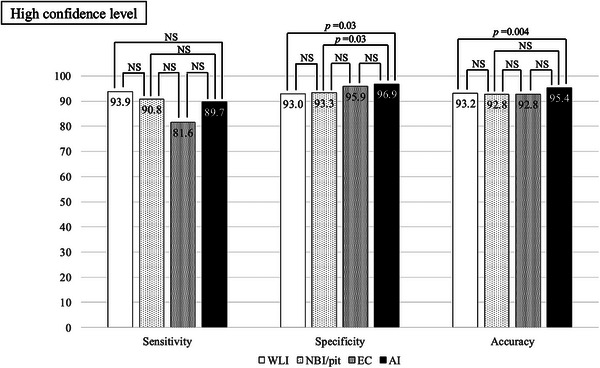
Sensitivity, specificity, and accuracy of lesion diagnosis with high confidence.

The endoscopists’ diagnoses were made with high confidence in 40.2% of lesions with WLI, 63.3% with magnifying endoscopy, 71.7% with EC, and 75.5% with CADx. When restricted to lesions for which the endoscopist's confidence was high, specificity differed significantly only when comparing magnifying endoscopy with CADx and when comparing WLI with CADx (Figure 3).

**FIGURE 4:**
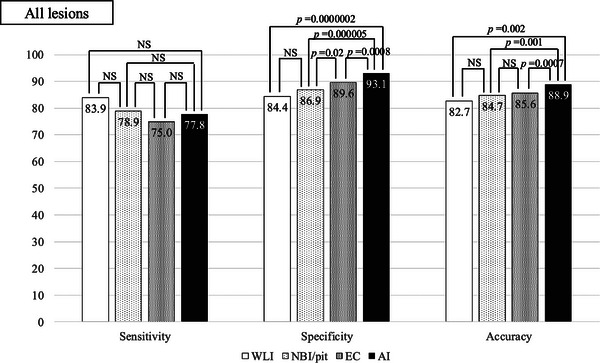
Sensitivity, specificity, and accuracy of all lesions.

**FIGURE 5:**
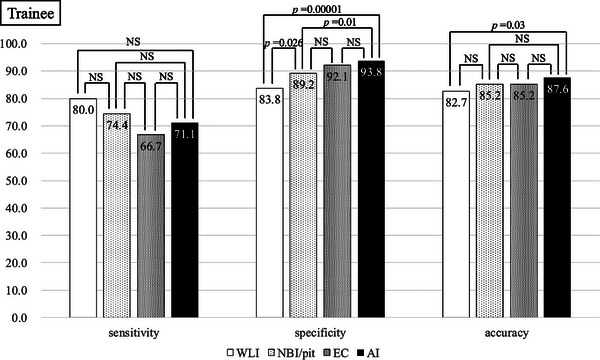
Sensitivity, specificity, and accuracy of lesions diagnosed by a trainee.

**FIGURE 6:**
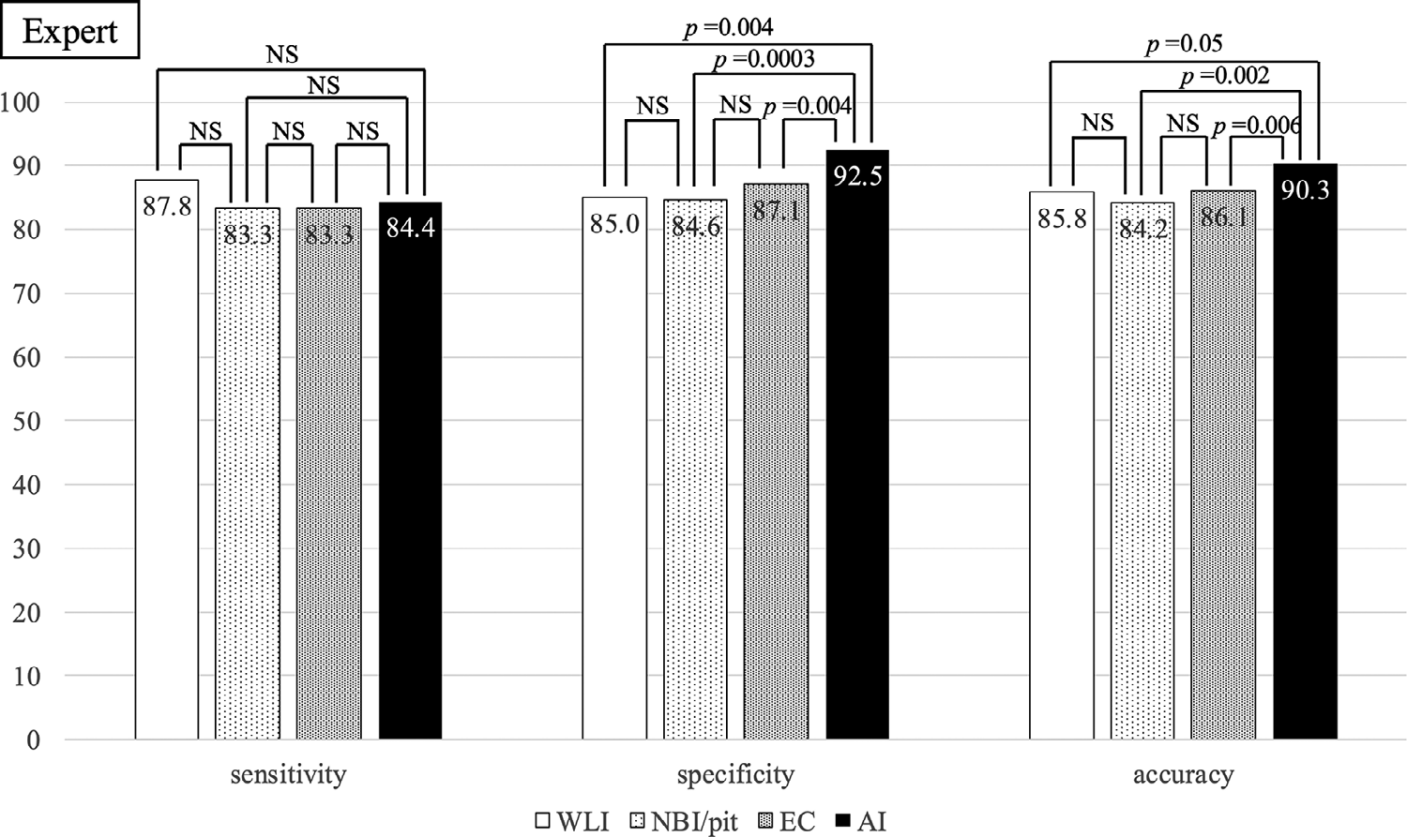
Sensitivity, specificity, and accuracy of lesions diagnosed by experts.

Figure 4 shows the diagnostic performances of trainee and expert endoscopists. There were no significant differences in sensitivity among trainees. Among the trainees, specificity differed significantly both between magnifying endoscopy and EC and between EC and WLI. The accuracy of the trainees differed significantly between CADx and WLI (Figure 5). Among the experts, CADx showed the highest specificity and accuracy. Other aspects of the performances of the experts did not differ significantly (Figure 6).

Table 3 shows that the diagnostic performances of trainee endoscopists were highly confident. Trainee endoscopists were highly confident of 31.5% of diagnoses made by WLI, 60.6% of those made by magnifying endoscopy, 68.2% of those made by EC, and 71.5% of those made by CADx. There were no significant differences among the diagnoses made by WLI, magnifying endoscopy, EC, and CADx (*p* > 0.05, Fisher's exact test).

**TABLE 3:** Diagnostic performances of trainee endoscopists for high confidence.

*n* = 330	White light	NBI/pit	EC	AI	
High confidence [95% CI]	*n* = 104 (31.5%) [26.5–36.8]	*n* = 200 (60.6%) [55.1–65.9]	*n* = 225 (68.2%) [62.9–73.2]	*n* = 236 (71.5%) [66.3–76.3]	
Sensitivity (%) [95% CI]	91.7 [73.0–99.0]	86.5 [71.2–95.5]	77.8 [62.9–88.8]	84.8 [71.1–93.7]
Specificity (%) [95% CI]	91.3 [82.8–96.4]	92.6 [87.5–96.1]	96.7 [92.9–98.8]	96.8 [93.3–98.8]
Accuracy (%) [95% CI]	91.3 [84.2–96.0]	91.5 [86.7–95.0]	92.9 [88.7–95.9]	94.5 [90.8–97.0]

Abbreviations: AI, artificial intelligence; CI, confidence interval; EC, endocytoscopy; NBI, narrow‐band imaging.

Table 4 shows the diagnostic performances of which expert endoscopists were highly confident. Expert endoscopists were highly confident of 48.8% of diagnoses made by WLI, 66.1% of those made by magnifying endoscopy, 75.2% of those made by EC, and 79.4% of those made by CADx. There were no significant differences among the diagnoses made by WLI, magnifying endoscopy, EC, and CADx (*p* > 0.05, Fisher's exact test).

**TABLE 4:** Diagnostic performances of expert endoscopists for high confidence.

*n* = 330	White light	NBI/pit	EC	AI	
High confidence [95% CI]	*n* = 161 (48.8%) [43.3–54.3]	*n* = 218 (66.1%) [60.7–71.2]	*n* = 248 (75.2%) [70.1–79.7]	*n* = 262 (79.4%) [74.6–83.6]	
Sensitivity (%) [95% CI]	95.2 [83.9–99.4]	94.0 [83.5–98.7]	86.2 [74.6–93.9]	93.5 [84.3–98.2]
Specificity (%) [95% CI]	94.1 [88.3–97.6]	94.0 [89.3–97.1]	95.3 [91.2–97.8]	97.0 [93.6–98.9]
Accuracy (%) [95% CI]	94.4 [89.7–97.4]	94.0 [90.0–96.8]	93.1 [89.3–96.0]	96.2 [93.1–98.2]

Abbreviations: AI, artificial intelligence; CI, confidence interval; EC, endocytoscopy; NBI, narrow‐band imaging.

Among 660 images diagnosed by 10 endoscopists, 270 images showed low confidence with WLI and high confidence with AI. Of these, 220 images (81.5%) were correctly diagnosed initially and remained correct. Five images (2%) were initially correct but later misdiagnosed. Thirty‐five images (13%) were initially misdiagnosed but later correctly diagnosed, and 10 images (3.5%) remained misdiagnosed. Of the 35 images initially misdiagnosed but ultimately correctly diagnosed, the breakdown was as follows: five (14.3%) of T1b images, two (5.7%) of T1a images, 20 (57.1%) of Tis images, and eight (22.9%) of adenoma images. The interobserver agreement values were 0.614 (95% confidence interval [95%CI]: 0.578–0.650), 0.609 (95%CI: 0.573–0.645), 0.579 (95%CI: 0.543–0.615), and 0.700 (95%CI: 0.664–0.736) for WLI, magnifying endoscopy, EC, and CADx‐assisted, respectively.　As for intraobserver agreement, it has already been demonstrated in previous studies [14], and we believe there is no significant difference from those findings.

## 4 Discussion

In this study, we documented progressive improvement in the diagnostic accuracy of endoscopists for identifying T1b cancer when employing WLI, magnifying endoscopy, EC, and CADx in that order. As we progressed from WLI to AI, the low confidence rate decreased, but the overall accuracy during low confidence remained suboptimal (81.5%). CADx‐assistance achieved the best performance. Furthermore, we found a positive correlation between high confidence and diagnostic accuracy, especially when AI technologies had been utilized. The Kappa coefficient was Moderate up to WLI‐EC but is Substantial in AI, indicating a slight improvement in agreement in AI.

Assessment of early colorectal cancers is important because there is a relatively high risk that deep submucosal cancers will have produced lymph node metastases and therefore require surgical resection. However, it has been difficult to correctly assess whether or not these cancers have invaded the deep submucosal layer (T1b). Vleugels et al. [15] reported that, in Dutch screening programs making optical diagnoses, only 39% of T1 colorectal cancers were accurately diagnosed, leading to suboptimal treatment decisions. They noted that patients with misdiagnosed T1 colorectal cancers often required additional surgeries (41% vs. 11% for correctly diagnosed cases). In a study in Japan using magnifying chromoendoscopy, deep submucosal invasion was only identified with 74.2% sensitivity and 68.6% specificity [16]. Currently, magnifying chromoendoscopy is regarded as the gold standard for assessing depth of invasion because it is more accurate than NBI [17, 18]. EC, a recently developed tool, is able to visualize both structural and cellular atypia; hence, it has the potential to more accurately assess invasion depth than does magnifying chromoendoscopy [8].

In this study, it was found that specificity and accuracy improved when endoscopists were presented with magnifying endoscopy, EC, and AI, in that order (Figure 4). For both experts and non‐experts, specificity and accuracy were highest when the endoscopists based their diagnoses on AI output. (Figures 5 and 6) Lesions with high confidence on WLI accounted for 265 lesions (approximately 40%). The remaining approximately 60% were low confidence, indicating the need for examination using other modalities (NBI, pit, EC, etc.). It is possible that 40% of lesions with high confidence were typical lesions that were easy to diagnose. And in both trainees and experts, WLI achieves the highest sensitivity in high‐confidence cases (Tables 3 and 4). Regarding the reason for the high sensitivity in WLI, we believe that when evaluating with WLI, the desire to avoid missing invasive cancer leads to a high sensitivity and consequently lower specificity. In clinical practice, if cancer is not suspected with WLI, there is a risk of not proceeding to magnifying endoscopy or EC. Therefore, high sensitivity is considered natural. Sakamoto et al. showed that, while diagnoses made by using magnifying chromoendoscopy are more accurate than those made by conventional colonoscopy, less experienced endoscopists struggle to reliably estimate invasion depth [19]. These findings suggest that, regardless of years of experience, AI may aid in diagnosing the depth of early colorectal cancers. In the present study, the percentage of lesions diagnosed with high confidence by participating endoscopists increased as they progressed through the diagnostic modalities of WLI, magnifying endoscopy, EC, and AI. Confidence level is an important factor when diagnosing diminutive polyps because some diminutive polyps are missed when making pathological diagnoses [20]. The significance of the level of confidence when diagnosing colorectal cancer has not been reported previously. We believe that improving confidence levels is clinically meaningful because the determined depth of a lesion is directly linked to treatment strategy.

CADx systems show promise for enhancing diagnostic accuracy in endoscopy. However, the interaction between CADx systems and endoscopists, especially in clinical settings, is critical to their success. Djinbachian et al. reported that autonomous AI systems achieve higher concordance with surveillance intervals than do AI‐assisted human diagnostics: concordance rates were 91.5% for autonomous AI and 82.1% for AI‐assisted by human (*p* = 0.016) [21]. Zander et al. found that endoscopists often undervalue CADx assessments, limiting the improvement in making diagnoses that they can confer [22]. Similarly, Reverberi et al. noted that overconfidence on the part of endoscopists can hinder acceptance of CADx outputs [23]. They emphasized the need to optimize interactions between endoscopists and CADx to create effective hybrid teams. Displaying confidence scores for CADx assessments is one possible strategy for improving these interactions. Confidence scores can help endoscopists gauge the certainty of AI assessments. That the EndoBRAIN‐Plus system used in this study includes confidence scores may have contributed to our favorable outcomes. Zander et al. also suggested making use of CADx optional rather than mandatory [22]. This approach allows endoscopists to consult CADx only when uncertain, potentially increasing adoption of CADx without reducing confidence in their own ability. Such flexibility could enhance collaboration between human expertise and AI capabilities. Future research should analyze clinicians’ decision‐making processes in more detail and develop strategies for better integrating CADx into clinical workflows. This would facilitate these technologies reaching their full potential in clinical practice.

This study had some limitations. First, because it was a single‐center retrospective study, it had inherent limitations in patient selection, potentially leading to selection bias and limiting the generalizability of the findings. Second, the test images used in this study were selected by the researchers, also potentially introducing selection bias. Third, this study has a limited sample size. Fourth, we excluded SSLs with dysplasia (SSLDs) and SSLD‐derived invasive cancers. These limitations might be considered to affect the generalizability of the results. In contrast, a major strength of this study is its comprehensive evaluation of multiple diagnostic modalities, including WLI, magnifying endoscopy, EC, and AI, in a single study, enabling direct comparison of these modalities.

In conclusion, EC most frequently yielded high‐confidence diagnoses. The highest diagnostic accuracy was achieved when endoscopists used AI assistance. Understanding the unique characteristics of each diagnostic modality and combining them may enhance the accuracy of colorectal lesion depth assessment.

## Author Contributions

**Eri Tamura**: Data acquisition; data analysis and interpretation; statistical analysis; drafting the manuscript. **Shin‐ei Kudo**: Study supervision; critical revision; final approval of the manuscript. **Masashi Misawa**: Study conception and design; data acquisition; data analysis and interpretation; statistical analysis; drafting the manuscript. **Shunto Iwasaki, Shigenori Senba, Tomoya Shibuya, Shun Kato, Takanori Kuroki, Yuta Sato, Tatsuya Sakurai, Yushi Ogawa, Yuta Kouyama, Yasuharu Maeda, Katsuro Ichimasa, Noriyuki Ogata, Takemasa Hayashi, Kunihiko Wakamura,** and **Tetsuo Nemoto**: Data acquisition. **Toshiyuki Baba** and **Fumio Ishida**: Study supervision; auditing.

## Conflicts of Interest

Shin‐ei Kudo and Masashi Misawa received speaking honoraria from Olympus Corporation and have ownership interests in the products of Cybernet Systems. Shin‐ei Kudo and Masashi Misawa have patents (Japan Patent JP 6059271 and JP 6580446) licensed to Cybernet Systems and Showa University. Tetsuo Nemoto received a research grant from Olympus Corporation and Cybernet Systems for other studies. Masashi Misawa is one of the Associate Editors of the Digestive Endoscopy Journal. The other authors declare no conflicts of interest.

## Funding

This work was supported by JSPS KAKENHI Grant Number 23K02744.

## Ethics Statement

This study was approved by the Ethics Committee of Showa University (No. 22‐191‐B).

## Consent

N/A

## Clinical Trial Registration

N/A

## References

[1] L. H. Biller and D. Schrag , “Diagnosis and Treatment of Metastatic Colorectal Cancer: A Review,” JAMA 325 (2021): 669–685. 33591350 10.1001/jama.2021.0106

[2] S. Tanaka , H. Kashida , Y. Saito , et al., “Japan Gastroenterological Endoscopy Society Guidelines for Colorectal Endoscopic Submucosal Dissection/Endoscopic Mucosal Resection,” Digestive Endoscopy 32 (2020): 219–239. 31566804 10.1111/den.13545

[3] Y. Kobayashi , S. E. Kudo , H. Miyachi , et al., “Clinical Usefulness of Pit Patterns for Detecting Colonic Lesions Requiring Surgical Treatment,” International Journal of Colorectal Disease 26 (2011): 1531–1540. 21607587 10.1007/s00384-011-1246-0

[4] S. E. Kudo , K. Wakamura , N. Ikehara , et al., “Diagnosis of Colorectal Lesions With a Novel Endocytoscopic Classification—A Pilot Study,” Endoscopy 43 (2011): 869–875. 21837586 10.1055/s-0030-1256663

[5] M. Misawa , S. E. Kudo , Y. Takashina , et al., “Clinical Efficacy of Endocytoscopy for Gastrointestinal Endoscopy,” Clinical Endoscopy 54, no. 4 (2021): 455–463. 34233111 10.5946/ce.2021.165 PMC8357585

[6] A. Mitsala , C. Tsalikidis , M. Pitiakoudis , et al., “Artificial Intelligence in Colorectal Cancer Screening, Diagnosis and Treatment. A New Era,” Current Oncology 28 (2021): 1581–1607. 33922402 10.3390/curroncol28030149 PMC8161764

[7] I. Barua , P. Wieszczy , S.‐E. Kudo , et al., “Real‐Time Artificial Intelligence–Based Optical Diagnosis of Neoplastic Polyps During Colonoscopy,” NEJM Evidence 1 (2022): EVIDoa2200003. 38319238 10.1056/EVIDoa2200003

[8] S. E. Kudo , Y. Mori , K. Wakamura , et al., “Endocytoscopy Can Provide Additional Diagnostic Ability to Magnifying Chromoendoscopy for Colorectal Neoplasms,” Journal of Gastroenterology and Hepatology 29 (2014): 83–90. 23980563 10.1111/jgh.12374

[9] M. Ahadi , A. Sokolova , I. Brown , et al., “The 2019 World Health Organization Classification of Appendiceal, Colorectal and Anal Canal Tumours: An Update and Critical Assessment,” Pathology 53 (2021): 454–461. 33461799 10.1016/j.pathol.2020.10.010

[10] Y. Mori , S. E. Kudo , M. Misawa , et al., “Artificial Intelligence‐assisted Colonic Endocytoscopy for Cancer Recognition: A Multicenter Study,” Endoscopy International Open 9 (2021): E1004–E1011. 34222622 10.1055/a-1475-3624 PMC8211486

[11] P. Ponugoti , A. Rastogi , T. Kaltenbach , et al., “Disagreement Between High Confidence Endoscopic Adenoma Prediction and Histopathological Diagnosis in Colonic Lesions </= 3 Mm in Size,” Endoscopy 51 (2019): 221–226. 30722072 10.1055/a-0831-2348

[12] T. Khuc , A. Agarwal , F. Li , et al., “Accuracy and Inter‐observer Agreement among Endoscopists for Visual Identification of Colorectal Polyps Using Endoscopy Images,” Digestive Diseases and Sciences 68 (2023): 616–622. 35947305 10.1007/s10620-022-07643-0

[13] Y. Kanda , “Investigation of the Freely Available Easy‐to‐use Software ‘EZR’ for Medical Statistics,” Bone Marrow Transplantation 48 (2013): 452–458. 23208313 10.1038/bmt.2012.244 PMC3590441

[14] T. Kudo , K. Suzuki , Y. Mori , et al., “Endocytoscopy for the Differential Diagnosis of Colorectal Low‐grade Adenoma: A Novel Possibility for the “Resect and Discard” Strategy,” Gastrointestinal Endoscopy 91 (2020): 676–683. 31785276 10.1016/j.gie.2019.11.029

[15] J. L. A. Vleugels , L. Koens , M. G. W. Dijkgraaf , et al., “Suboptimal Endoscopic Cancer Recognition in Colorectal Lesions in a National Bowel Screening Programme,” Gut 69 (2020): 977–980. 31822579 10.1136/gutjnl-2018-316882 PMC7282551

[16] T. Shimura , M. Ebi , T. Yamada , et al., “Magnifying Chromoendoscopy and Endoscopic Ultrasonography Measure Invasion Depth of Early Stage Colorectal Cancer With Equal Accuracy on the Basis of a Prospective Trial,” Clinical Gastroenterology and Hepatology 12 (2014): 662–668.e1–2. 10.1016/j.cgh.2013.06.022 23872238

[17] T. Sakamoto , T. Nakajima , T. Matsuda , et al., “Comparison of the Diagnostic Performance Between Magnifying Chromoendoscopy and Magnifying Narrow‐band Imaging for Superficial Colorectal Neoplasms: An Online Survey,” Gastrointestinal Endoscopy 87 (2018): 1318–1323. 29309778 10.1016/j.gie.2017.12.021

[18] S. Kobayashi , M. Yamada , H. Takamaru , et al., “Diagnostic Yield of the Japan NBI Expert Team (JNET) Classification for Endoscopic Diagnosis of Superficial Colorectal Neoplasms in a Large‐scale Clinical Practice Database,” United European Gastroenterology Journal 7 (2019): 914–923. 31428416 10.1177/2050640619845987 PMC6683640

[19] T. Sakamoto , T. Matsuda , T. Nakajima , et al., “Impact of Clinical Experience on Type V Pit Pattern Analysis Using Magnifying Chromoendoscopy in Early Colorectal Cancer: A Cross‐sectional Interpretation Test,” BMC Gastroenterology [Electronic Resource] 14 (2014): 100. 24885943 10.1186/1471-230X-14-100 PMC4046150

[20] D. K. Rex , C. Kahi , and M. O'Brien , “The American Society for Gastrointestinal Endoscopy PIVI (Preservation and Incorporation of Valuable Endoscopic Innovations) on Real‐time Endoscopic Assessment of the Histology of Diminutive Colorectal Polyps,” Gastrointestinal Endoscopy 73 (2011): 419–422. 21353837 10.1016/j.gie.2011.01.023

[21] R. Djinbachian , C. Haumesser , M. Taghiakbari , et al., “Autonomous Artificial Intelligence vs Artificial Intelligence‐Assisted Human Optical Diagnosis of Colorectal Polyps: A Randomized Controlled Trial,” Gastroenterology 167 (2024): 392–399.e2. 38331204 10.1053/j.gastro.2024.01.044

[22] Q. E. W. van der Zander , R. Roumans , C. H. J. Kusters , et al., “Appropriate Trust in Artificial Intelligence for the Optical Diagnosis of Colorectal Polyps: The Role of Human/Artificial Intelligence Interaction,” Gastrointestinal Endoscopy 100 (2024): 1070–1078.e10. 38942330 10.1016/j.gie.2024.06.029

[23] C. Reverberi , T. Rigon , A. Solari , et al., “Experimental Evidence of Effective Human‐AI Collaboration in Medical Decision‐making,” Scientific Reports 12 (2022): 14952. 36056152 10.1038/s41598-022-18751-2 PMC9440124

